# Noise-Resilient Acoustic Low Energy Beacon for Proximity-Based Indoor Positioning Systems

**DOI:** 10.3390/s21051703

**Published:** 2021-03-02

**Authors:** Teodoro Aguilera, Fernando J. Aranda, Felipe Parralejo, Juan D. Gutiérrez, José A. Moreno, Fernando J. Álvarez

**Affiliations:** Sensory Systems Research Group, University of Extremadura, 06006 Badajoz, Spain; fer@unex.es (F.J.A.); felipe@unex.es (F.P.); andy@unex.es (J.D.G.); josan@unex.es (J.A.M.); fafranco@unex.es (F.J.Á.)

**Keywords:** acoustic beacon, proximity-based positioning, location based services

## Abstract

Proximity-Based Indoor Positioning Systems (PIPSs) are a simple to install alternative in large facilities. Besides, these systems have a reduced computational cost on the mobile device of those users who do not continuously demand a high location accuracy. This work presents the design of an Acoustic Low Energy (ALE) beacon based on the emission of inaudible Linear Frequency Modulated (LFM) signals. This coding scheme provides high robustness to in-band noise, thus ensuring a reliable detection of the beacon at a practical range, after pulse compression. A series of experimental tests have been carried out with nine different Android devices to study the system performance. These tests have shown that the ALE beacon can be detected at one meter distance with signal-to-noise ratios as low as −12 dB. The tests have also demonstrated a detection rate above 80% for reception angles up to 50° with respect to the beacon’s acoustic axis at the same distance. Finally, a study of the ALE beacon energy consumption has been conducted demonstrating comparable power consumption to commercial Bluetooth Low Energy (BLE) beacons. Besides, the ALE beacon search can save up to 9% more battery of the Android devices than the BLE beacon scanning.

## 1. Introduction

Commonly, people find it challenging to navigate through shopping malls, hospitals, airports, or other large and busy buildings, even when signage and static maps are provided. This circumstance, together with the increasing implementation of technology in our lives, implies that more and more users demand an application that allows them to be guided through these complex indoor facilities. Many Indoor Positioning Systems (IPSs), whose development has significantly increased in the last two decades [1,2,3], strive to locate the user at all times and as accurately as possible. Different technologies such as Wi-Fi [4,5], Bluetooth Low Energy (BLE) [6,7], visible light [8,9] and ultrasound [10,11] have been extensively used in combination with positioning techniques that include triangulation, trilateration, multilateration, and fingerprinting, among others. Nevertheless, although such positioning can have many applications, in the vast majority of cases, users only need to know with some accuracy where they are and how to get to the desired Point of Interest (PoI). Moreover, these continuous and precise IPSs are difficult and expensive to implement in such complex environments and entail a high computational cost for the users’ smartphones [12].

Proximity-Based Indoor Positioning Systems (PIPSs) have generally been used to position people indoors in a practical and straightforward way. These systems have usually been implemented using technologies such as Radio Frequency Identification (RFID) [13,14], Near-Field Communication (NFC) [15], Quick Response (QR) codes [16] or BLE [17,18]. However, RFID, NFC, and QR codes, in addition to requiring the user’s active participation by bringing the reader very close to the tag, may not be feasible for visually impaired users. With respect to BLE technology, it has many applications in this field but also important disadvantages. First, this technology does not often provide the required accuracy [19]. Second, although BLE beacons have a low power consumption that allows them to operate for years, the cell phones’ energy consumption to scan them is very high [20]. Finally, the detection of BLE beacons may have discovery latencies of up to 10 s [21].

Acoustic technology has features that make it suitable for PIPS. First, ultrasonic positioning systems are a classical and reliable solution characterized by robust signals with coverage ranges of up to some tens of meters [22]. This robustness is provided by incorporating broadband signaling and the pulse compression detection technique, which ensure high resistance to in-band noise. Second, the mechanical nature of acoustic waves imposes the signal’s confinement, preventing false detections of beacons in adjacent rooms. Third, the transducers’ emission cone (directivity pattern) can be easily adjusted as a function of the acoustic waves’ frequency, thus achieving a precise directionality in the emission. This work proposes the design of a new Acoustic Low Energy (ALE) beacon and the corresponding signal detection algorithms that, once installed in the user’s smartphone, transform this device into the portable receiver of a PIPS that overcomes the problems mentioned above. This ALE beacon is based on a chirp-encoding scheme that allows its accurate identification by moving receivers with very low power (inaudible) emissions. Other encoding schemes, based on the use of pseudorandom codes, could be used to obtain a similar robustness to noise even with shorter emissions [23,24], but the matched filtering detection of these signals is not as resilient to Doppler shift as that of chirp signals [25,26], which would prevent their reliable detection in motion.

The ALE beacon is also equipped with a passive infrared (PIR) sensor that enables these emissions only when the user is detected in its surroundings, notably reducing the power consumption to save the battery’s life. As small as a matchbox, it can be strategically and seamlessly installed on walls, columns, ceilings, or stairs to help recognize and find specific PoIs.

The rest of the paper is organized as follows. Section 2 presents a comprehensive review of recent works directly related to the development of PIPS. In Section 3, the ALE beacon and corresponding signal detection algorithms are described in detail. Next, in Section 4, some experimental results are provided to analyze the system performance when using different devices as the proposed PIPS’s portable receiver. Finally, Section 5 presents the main conclusions that can be drawn from these results.

## 2. Related Works

The development of PIPS has been fostered by the smartphone appearance and its later evolution, due to the many technologies and embedded sensors that these devices include [27]. One example of these technologies are short-range radio frequency networks. Edwan et al. [28] integrated NFC proximity sensors with smartphones’ inertial sensors to provide navigation within indoor environments. Similarly, Ozdenuzci et al. [15] implemented an NFC proximity system to display location information for a user carrying a smartphone. A similar design was followed by Pecchioli et al. using QR codes [29]. Despite having a 100% accuracy detection rate, NFC and QR proximity systems require the user to be very close to the transmitter, usually in the range of centimeters, which is impractical for most real applications [30]. RFID has a more extensive communication range than NFC and has also been explored in this field. For example, Montaser and Moselhi [14] proposed a location identification system for construction projects. Their system operates with RFID for detecting the proximity of works and material, achieving a 100% accuracy in this detection. Tesoriero et al. [31] implemented an RFID proximity-based application to display information about pieces in art galleries and museums automatically. Nevertheless, its range is too short compared with other radio frequency technologies, and it is not incorporated in most off-the-shelf smartphones, so extra hardware is needed in the receiver for its deployment.

Wireless Local Area Networks (WLANs) are currently the most adopted technology for proximity-based applications [32]. Amutha and Nanmaran [33] designed and tested a proximity localization system to assist impaired persons using ZigBee transmitters incorporated into smart-home devices. The use of Wi-Fi access points for proximity applications has also been studied in [19,34], but it is somehow inefficient due to the protocol design and the spatial constraints of its transmitters. BLE is currently the preferred technology in this field due to its low power consumption design and scalability. Mackey et al. [35] built a smart parking system that assists drivers in finding empty lots and their cars’ position. Spachos et al. [36] built a BLE proximity location system to assist visitors in museums and exhibits. The system can also be used to detect behavior patterns in the visitors by museum administrators. Ceron et al. [37] used BLE beacons to monitor sedentary behaviors in in-home environments, correctly identifying the user activities and detecting continuously repeated sedentary behaviors. All these systems, and those based on ZigBee and Wi-Fi, used the Receive Signal Strength Indicator (RSSI) to indicate proximity. As it is well known, RSSI-based applications have several drawbacks since these measurements are unstable, strongly time-variant, and deeply affected by signal propagation effects in indoor environments and the presence of people in them.

More recently, LED light has also been used for proximity-based applications. These systems can use current infrastructure instead of adding a new one. Xie et al. [38] proposed an identification system for proximity in indoor environments using modulated LED and CMOS cameras. This system has a detection rate above 90% for distances below 6 m from the light source, but it is highly dependent on each device’s camera, which is not standardized, as is the case with Wi-Fi and BLE. A similar design using Manchester encoding for the LED light was used by Kim et al. [39] for guidance in museums.

Some works can also be found in the literature that propose using acoustic technology in the design of PIPS. One of the earliest designs [40] proposes a wireless wearable device capable of acquiring and identifying the coded signals sent by a set of ultrasonic beacons located at known positions. These beacons emit 16-bit sequences using a 40 kHz pulse modulated with an on/off keying scheme. For this purpose, several Xilinx XC40 FPGAs are also employed to process the signals received by specific ceramic transducers. The main disadvantage of this system is the need for a specifically designed receiver that severely limits its practical implementation.

A different approach is proposed in [41], where an Android device (Samsung Galaxy II) is used to run an application called RoomSense. This application uses the Fingerprinting (FP) technique based on the extraction of impulsive response features from measurements taken over 22 rooms in which 67 measurements are taken over different points inside each room. These measurements form the FP database with 5360 impulsive response measurements, which will be later cross-checked with the smartphone’s acquisitions during the positioning phase. The system is tested identifying the room in less than one second and reaching a success rate of more than 98% and 96% in the correct determination of the absolute position inside the room. The main drawback is the laborious process of generating the database that involves the FP technique’s offline phase in each of the rooms. Besides, this database must be updated from time to time as the acoustic parameter measurements may vary due to changes in the environment. A similar system called SoundLoc that also provides room-level positioning by identifying the Room Impulsive Response (RIR) is proposed in [42]. In this system, Maximum Length Sequences (MLSs) are emitted, and a Noise Adaptive Reverberation Extraction (NAER) algorithm is used to obtain the optimal feature extraction that enables room classification using artificial intelligence. The main shortcomings of this system are, once again, the need for a training phase before classification and the use of audible signals that can be annoying for users.

The previous work more closely related to the one presented here is described in [43]. Here, the authors propose the use of a mobile phone to identify rooms by detecting chirp-encoded ultrasonic signals. However, this system has important shortcomings that affect its performance and make it very difficult to implement in a practical application. First, it uses a bit-coding frequency band between 20 and 22 kHz. Since mobile phone microphones are designed to work in the audible range, there are many devices whose audio acquisition system imposes a sharp attenuation beyond 20 kHz and could not then be used as receivers. Second, the bandwidth allocated for each chirp is only 500 Hz. This narrow bandwidth requires a long emission duration to guarantee the minimum Time-Bandwidth Product (TBP) that ensures the correlation peak’s unambiguous identification [44]. This duration is a significant limitation when increasing the number of bits of the emission since it causes an excessive delay in the decoding process. Additionally, the time elapsed between transmissions should be increased to avoid multipath problems derived from long emissions [45]. A final weakness of this system is the lack of a strategy to reduce the acoustic beacons’ energy consumption, making it impractical to power them with batteries and thus limiting the portability and scalability of the whole proposal.

## 3. System Description

This work proposes the use of an acoustic beacon specifically designed to be used in PIPS. This section describes the hardware used in its design, along with its most relevant characteristics. A detailed explanation of the emission architecture and the signal processing carried out in the user’s smartphone is also provided.

### 3.1. Transmitter Module

**Figure 1:**
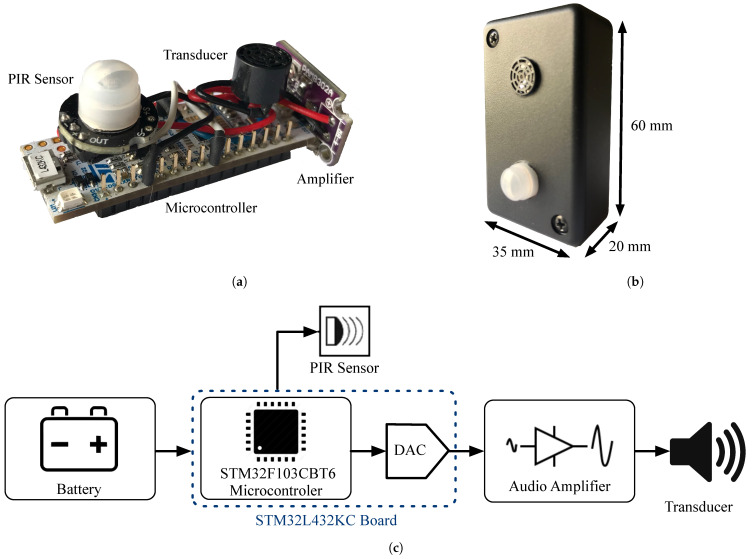
Acoustic beacon. (**a**) Hardware components. (**b**) Complete assembly. (**c**) Operating diagram.

Figure 1 shows the ultrasonic beacon developed in this work. The beacon is composed of a NUCLEO-L432KC [46] board, a PAM8302 Class D audio amplifier [47], a MH-SR602 Passive InfraRed (PIR) movement sensor [48] and an ultrasonic transducer [49]. Figure 1a shows the assembly of all these hardware components, and to the right, Figure 1b depicts the complete beacon with its plastic housing. Figure 1c below shows the functional block diagram of the acoustic beacon. Firstly, the board microcontroller is responsible for generating the coded acoustic emissions, for which it uses one of its two Digital to Analog Converters (DACs). These emissions are generated provided that the PIR sensor has detected movement within a 3 m range around the beacon. Once the emissions have been generated, their power is regulated by an audio amplifier. Subsequently, these signals are synthesized by the ultrasonic transducer. Finally, if the PIR sensor stops detecting movement for 5 s, the emission ends and will not resume until the infrared sensor is activated again.

In terms of beacon power supply, the selected microcontroller board offers several alternatives. Firstly, it can be powered by a 5 V source or battery through its micro USB port. It also has a 5 V input that allows the use of this voltage through one of its pins. Besides, it has another pin for 3.3 V voltage that requires the opening of two soldering bridges that make it impossible to reprogram the device later on. Finally, it has a Vin pin for voltages between 7 and 12 V, used in this assembly. A 9 V battery is attached to the beacon plastic housing and connected to the microcontroller through the *Vin* pin to perform the experimental tests. Note that it would also have been possible to connect it directly to the mains with the help of a small 12 V AC/DC converter.

Because the signals emitted are intended to be acquired by smartphones, it will be necessary to use frequencies below 20 kHz. Smartphone microphones and audio acquisition systems are designed for the audible range, and most of them sharply cut all those frequencies above 20 kHz. This limit can vary slightly by 1 or 2 kHz, depending on the manufacturer. In this work, a piezoelectric transducer with a maximum response frequency of 40 kHz has been chosen, which can also reliably operate in the high-frequency acoustic band.

**Figure 2:**
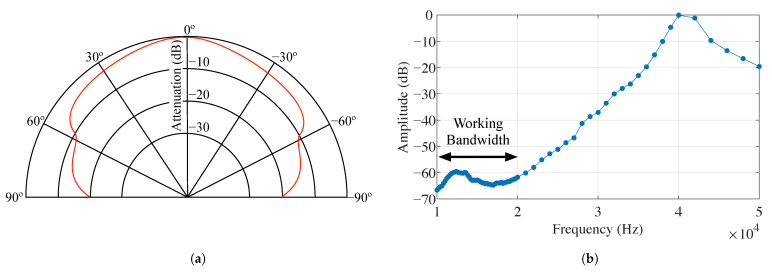
Acoustic transducer features. (**a**) Acoustic transducer directivity pattern [49]. (**b**) Acoustic transducer frequency response.

Figure 2 shows the main features of this transducer. On one left hand side, the directivity pattern supplied by the manufacturer is shown, in which a 120° emission cone amplitude is observed without significant attenuation of the sound power level. Since the manufacturer’s tests have been performed at the transducer’s resonance frequency (40 kHz), it is expected that the emission lobe width will be even greater at frequencies below 20 kHz. On the other side, Figure 2b shows the transducer frequency response. Since the manufacturer only provides data between 30 kHz and 50 kHz, it has been necessary to evaluate this response between 10 kHz and 50 kHz experimentally. This study has been conducted using a high-sensitivity microphone [50] connected to an amplifier module [51] with 50 dB gain. The transducer is connected to an arbitrary waveform generator [52] which generates sinusoidal signals over the entire frequency range of interest. Amplitude measurements are taken every 200 Hz for frequency values between 10 kHz and 20 kHz. For frequency values above 20 kHz and up to 40 kHz, measurements are made every 1 kHz, and from then on, up to 50 kHz, every 2 kHz. These measurements have been taken in a clear room with low ambient noise and analogously to the manufacturer’s procedure, taking separation of 0.3 m between transmitter and receiver.

As shown, the emitted signals present a 60 dB attenuation in the working bandwidth, relative to the expected amplitude at the resonance frequency. In contrast, the audio amplifier, according to the manufacturer’s specifications, provides a gain of 25 dB with a Total Harmonic Distortion (THD) below 1%, giving quality amplification in the 10–20 kHz audible frequency range. In any case, the emitted Sound Pressure Level never exceeds 40 dB of measured ambient noise. These emissions are inaudible to adults but can be slightly perceived at very close range by teenagers.

### 3.2. Signal Coding

As stated before, one of the main features of the ALE beacon is the very low power of its inaudible emissions. To ensure the reliable detection of this beacon at a practical range, we have implemented a signal coding scheme capable to provide enough processing gain after pulse compression. This technique is commonly used in radar and sonar systems to improve the Signal to Noise Ratio (SNR) of the receptions and to increase the resolution of distance measurements. Pulse compression is appropriate on signals with good auto-correlation properties and robust against Doppler shifts. One of the signals that better meet these requirements are the Linear Frequency Modulated (LFM) waveforms, commonly known as chirps [26].

A linear chirp of unit amplitude can be defined as:

(1) $$w\left(t\right)=\exp\left[i2\pi \left(f_{0}t+\frac{B}{2T}t^{2}\right)\right]$$

where $f_{0}$ is the chirp center frequency, *T* is the chirp duration time and *B* the chirp bandwidth defined between $f_{1}$ and $f_{2}$. For a chirp with a bandwidth *B*, emitted for *T* seconds, a compression gain equal to its Time-Bandwidth Product (TBP) can be obtained. Additionally, a signal amplification equal to $\sqrt{\left(T\times B\right)}$ is achieved with a measured pulse width of 1/B at −4 dB from its peak [53].

**Figure 3:**
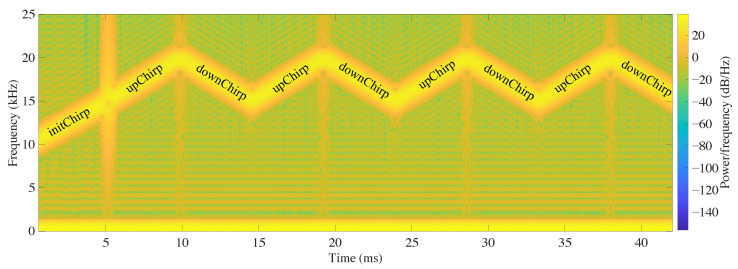
Spectrogram of the emitted signal coded as 10101010.

In this work, chirps signals are proposed to modulate an n-bit binary code. In particular, we have used n=8 bits to identify up to 256 beacons. To do this, chirps increasing linearly in frequency between 15 and 20 kHz (upChirps) are used to code the ones, and chirps decreasing linearly between 20 and 15 kHz (downChirps) to code the zeros. Despite sharing the same frequency range, these chirps offer almost orthogonal cross-correlations properties since their frequency sweeps slopes are opposite. A synchronism signal is also necessary to indicate the emission beginning, for which an upchirp with frequencies between 10 and 15 kHz has been used. Hence, nine chirps (1 synchronism chirp + 8 binary coding chirps) are included in each emitted message, with no gaps between consecutive sendings. As these signals are acquired and processed by a smartphone, a total length of 4096 samples has been selected. This size (4 kB) offers optimal performance in the smartphone’s internal memory allocation. Therefore, a length of 496 samples was chosen for the initChirp and of 450 samples for each of the upChirps and downChirps that compose the beacon’s binary identification code, thus giving a total length of 496+(8×450)=4096 samples. These signals are sampled by the 12-bit microcontroller’s DAC at a 96 kS/s rate, which means that the total duration of the message is 0.0052+(8×0.0427)=0.0427 s. As an example, Figure 3 shows the spectrogram of an emitted signal that has been coded as 10101010. Note that, since the chirp processing gain is given by $\sqrt{\left(T\times B\right)}$, it is estimated that the peak amplitude of the compressed pulse would have a minimum amplification factor of 4.85, equivalent to 13.71 dB.

### 3.3. Receiver Module and Signal Analysis

The receiver module has been programmed for Android devices. This platform has been adopted because it has 86% of the world’s cell phone market share. However, there is a wide diversity of Android devices that also incorporate very heterogeneous components. Regarding this work interest, the most relevant factor is the lack of homogeneity when managing the microphones integrated in the smartphones. Depending on the mobile phone brand and model, the manufacturer incorporates microphones of different qualities. Generally, high-end phones have two or more good quality microphones located at the bottom, top, and even at the smartphone’s back. Generally, the lower microphone is for calling conversations, the upper one is for filtering ambient noise, and the rear one (next to the camera) is for video recording. However, mid and low-range phones usually have a lower microphone of acceptable quality, and not always a microphone at the top; in which case, it is generally of low quality. In all these cases (high or low-middle quality phones), it is up to the manufacturer to decide whether to allow the programmer access to these microphones.

In this work, the acquisition and signal processing are made entirely in the smartphone. For signal acquisition, the AudioRecord [54] class has been used, which allows recording audio in Pulse Code Modulation (PCM) format with 16-bit resolution. The acquisition sample rate has been set to 96 kS/s, and the buffer size has been established in 4096 samples. To the authors’ knowledge, Android does not include any specific Digital Signal Processing (DSP) library that allows direct signal convolution or correlation, so the open jTransforms library [55] has been used. This library performs the Fast Fourier Transform (FFT) to carry out the signal processing in the frequency domain. Once the signal is processed, it is returned to the time domain using its Inverse Fast Fourier Transform (IFFT) function.

**Figure 4:**
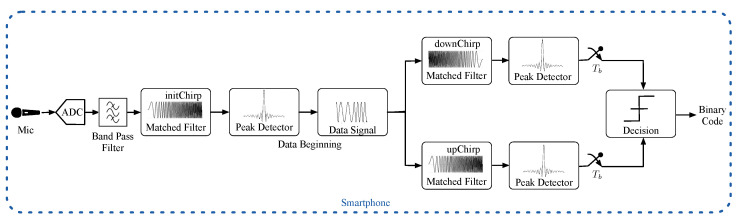
Diagram of the signal processing carried out in the smartphone.

**Figure 5:**
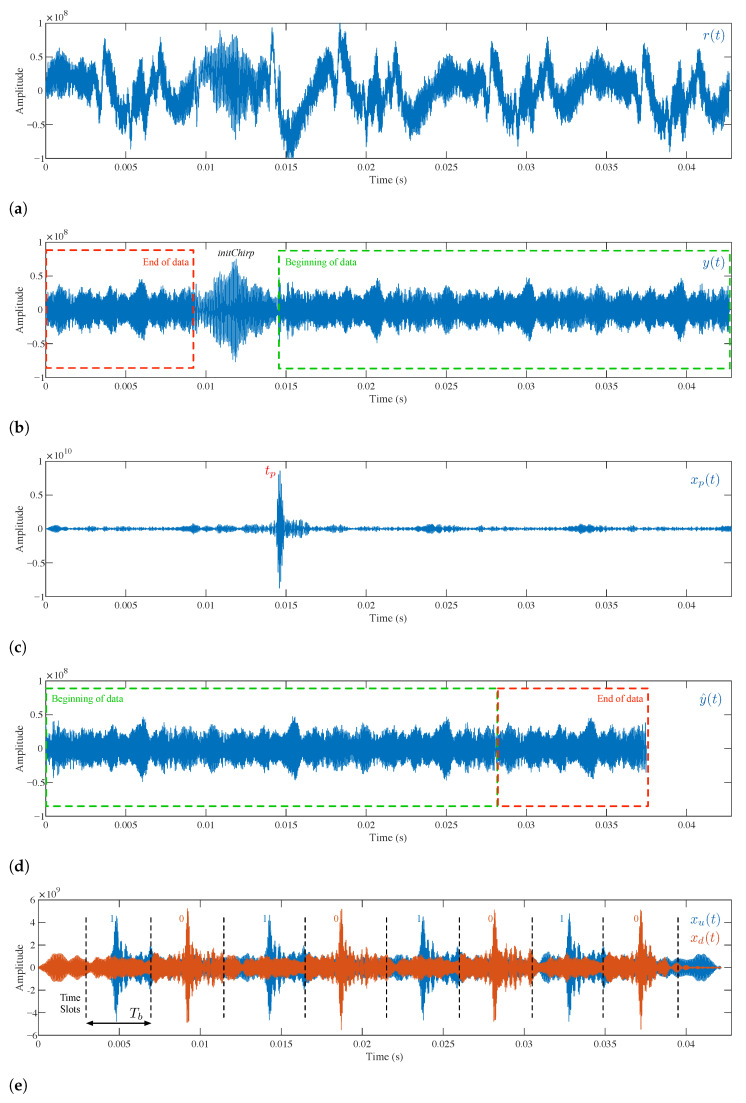
Receiver signal processing. (**a**) Raw received signal. (**b**) Pass band filtered received signal. (**c**) Correlation with initChirp. (**d**) Rearranged data signal. (**e**) Correlation with upChirp and downChirp.

Figure 4 contains a schematic of the signal processing carried out in the smartphone:

As shown in this figure, the smartphone acquires the acoustic signal through its embedded microphone. This signal is digitized with 16-bit resolution by the phone’s Analog-to-Digital Converter (ADC), see Figure 5a. Next, a bandpass filtering between 10 and 20 kHz is performed to clean out noisy signals, see Figure 5b. In particular, a Finite Impulsive Response (FIR) filter with 216 coefficients has been designed for this purpose. This filter b(t) is convolved with the received signal r(t) in the frequency domain, i.e.,:

(2) $$F\left[b(t)*r(t)\right]=B\left(s\right)\cdot R\left(s\right)$$

where the F operator denotes FFT and the ∗ symbol is the convolution operation. The frequency domain convolution is the product of the two signal transforms. This product, of complex numbers, results in the filtered signal Y(s), which has to be returned to the time domain through an IFFT, so:

(3) $$F^{-1}\left[B(s)\cdot R(s)\right]=F^{-1}\left[Y(s)\right]=y\left(t\right)$$

being $F^{-1}$ the IFFT operator and y(t) the filtered signal in the time domain. Note that the FIR filter introduces a delay of (N−1)/2 samples in the filtered signal, being *N* the filter order. This delay is compensated for by adding zeros to the end of the signal received before it is convolved.

Since the signal emission is continuous, it allows for asynchronous signal detection. However, the message start can be located at any time within y(t). To find this point, y(t) is correlated with the initChirp pattern p(t). By obtaining the maximum value of the correlated signal, it is possible to determine the instant within y(t) where the beacon identification data starts. This correlation is also performed in the frequency domain by computing the FFT of the time-reversed initChirp, and then multiplying the resulting complex signal by the FFT of y(t), i.e.,:

(4) $$F\left[p(t)⊗y(t)\right]=F\left[p(-t)*y(t)\right]=P\left(-s\right)\cdot Y\left(s\right)=X_{p}\left(s\right)$$

where ⊗ denotes the correlation operation and $X_{p}\left(s\right)$ represents the correlated signal in the frequency domain. The correlated signal in the time domain, represented in Figure 5c, can be easily obtained by computing the IFFT of $X_{p}\left(s\right)$:

(5) $$x_{p}\left(t\right)=F^{-1}\left[X_{p}\left(s\right)\right]$$

The beacon identification information starts within y(t) at the instant when the maximum value of $x_{p}\left(t\right)$ is observed:

(6) $$t_{p}=\underset{t}{\mathrm{argmax}}\;\;\left[x_{p}\left(t\right)\right]$$

Once the data starting instant $t_{p}$ has been located, it is necessary to reorder y(t) to decode the binary message correctly. The signal portion from $t_{p}$ to the end of the buffer is placed at the beginning of the new sorted signal $\hat{y}\left(t\right)$. Then the fragment from the beginning of the buffer to $t_{p}$ minus the duration of the initChirp signal is appended at the end of the previous portion. Figure 5d details the filtered signal reorganization.

Next, the signal $\hat{y}\left(t\right)$ is correlated with the upChirp and downChirp patterns to obtain the correlated signals $x_{u}\left(t\right)$ and $x_{d}\left(t\right)$, following a similar procedure to the one described above (Equations (4) and (5)). As depicted in Figure 5e, each one of these correlations is divided into eight time slots of duration $T_{b}$, $x_{u}^{i}\left(t\right)$ and $x_{d}^{i}\left(t\right)$ with i=1,⋯,8. Then, the maximum value of each $x_{u}^{i}\left(t\right)$ slot is compared with the maximum value of the corresponding $x_{d}^{i}\left(t\right)$ slot to decide whether this slot is decoded as a 1 or a O:

(7) $$\mathrm{bit}\;\left(i\right)=\left\{\begin{array}{cc} 1 & \mathrm{if}\;\;\max\left(x_{u}^{i}\left(t\right)\right)>\max\left(x_{d}^{i}\left(t\right)\right)\\ 0 & \mathrm{if}\;\;\max\left(x_{u}^{i}\left(t\right)\right)<\max\left(x_{d}^{i}\left(t\right)\right) \end{array}\right.\;\mathrm{for}\;\;i=1,\cdots ,8$$

These eight bits allows the receiver to identify a total of $2^{8}=256$ different beacons, although this number can be easily increased by adding more upChirps or downChirps to the limit where the processing time of the received signal is acceptable. The number of bits can also be increased by reducing the chirps’ length to limits where the detection is still reliable. Obviously, both techniques could be combined to increase the number of beacons to identify without compromising the system performance.

## 4. Experimental Results

This section shows the experimental results obtained in the evaluation of the proposed system. A system characterization was carried out to analyze noise tolerance and sensing coverage. Next, the system performance in a real environment was evaluated by deploying a set of 18 beacons inside an office building. Finally, the energy consumption of both the beacon and different receivers was assessed. In particular, nine Android devices (seven smartphones and two tablets) covering different brands and quality ranges, were used to highlight the differences in performance that could be found within the wide range of devices available on the market. These devices were a Xiaomi Mi 10 Pro, a Xiaomi Redmi Note 8 Pro, a Xiaomi Mi A3, a Huawei P30, an Elephone P9000, a BQ Aquaris X, a Samsung Galaxy Galaxy J5, a Samsung Galaxy Tab S5e and a Samsung Galaxy Tab S6.

### 4.1. System Characterization

The system characterization was carried out by measuring its availability. System availability was defined as the percentage of successful detections obtained at a certain point where the smartphone or tablet was evaluated. In that sense, it should be noted that the variation in system availability could be produced by the deterioration or attenuation of the signal received.

Regarding the received signal deterioration, various causes such as interference, multipath, the Doppler effect, or in-band noise can affect it. Due to the vast field of study involved in addressing all these phenomena, the authors have focused on evaluating only the consequences of the most determining factor for this particular system: in-band noise. Other causes, such as the Doppler effect, multipath, or interferences, although they are of great importance in other accurate acoustic IPS, are not crucial in the proximity-based positioning approach used in this work.

On its side, the received signal attenuation was mainly due to three factors. The first one was the distance between the beacon and the mobile device that could also be accentuated to a greater or lesser extent by the emitted signal frequency. The second attenuation factor of the received signal was the combined effect of the beacon’s transducer and device’s microphone directivity patterns. The directivity pattern of the emitting beacon was supplied by the manufacturer and included in Figure 2a. However, none of the smartphone or tablet manufacturers offered these data. Knowing this microphone’s information would have been useful to explain the attenuation suffered in different orientations between emitter and receiver. Unfortunately, the authors of this paper do not have the technical means (anechoic chamber and high bandwidth transducer), enabling them to obtain reliable directivity patterns of these device microphones. Finally, the third factor influencing the received signal’s amplitude was the beacon battery level. For these tests, the beacon was connected to the mains using a 5 V cell phone charger. This constant power supply ensured a fair comparison between all devices.

All tests carried out to characterize the system were conducted in a clear room of dimensions 6.17×4.82×3.12 m${}^{3}$, with a concrete ceiling, tile floor, two plasterboard walls and two glass walls (the one facing the emitter covered with an acoustic curtain). The T60 reverberation time of this room was 1.16 s according to Sabine’s formula. However, this time was reduced to 0.57 s when using the Allen and Berkley’s Image Method [56] to obtain the acoustic impulse response induced by the emitter at the exact location of the receiver.

#### 4.1.1. Noise Tolerance

**Figure 6:**
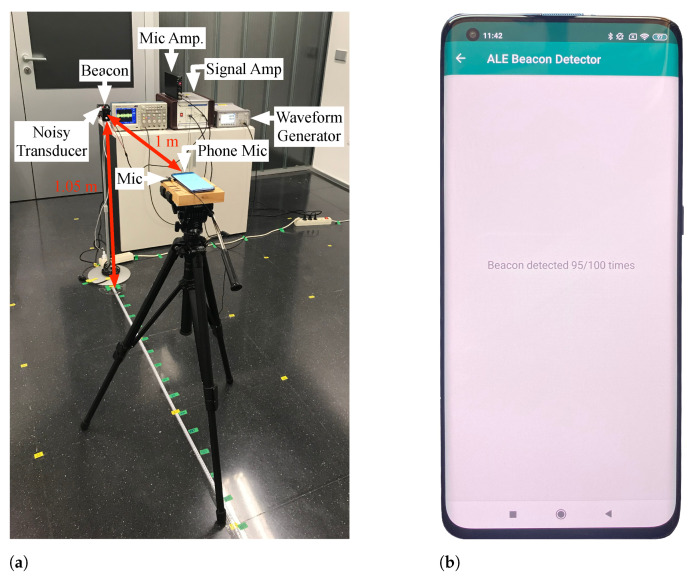
Noise tolerance experiment. (**a**) Experimental setup. (**b**) Android application interface.

The evaluation of the system’s performance facing in-band noise addition was done with the experimental setup illustrated in Figure 6a. First, using the ultrasonic microphone, its amplifying stage, and the oscilloscope [57], the emitted signal amplitude was measured at a 1-m distance. According to this amplitude, the arbitrary waveform generator produced 100 kHz bandwidth Additive White Gaussian Noise (AWGN) signals to obtain Signal to Noise Ratios (SNR) ranging from 0 dB to −12 dB. An identical transducer to the one used for the ultrasonic beacons synthesized the noise signals. The noising transducer was coupled to the beacon, ensuring the same emitted signal power at a meter distance and the same frequency response for both emitters.

Moreover, an Android application was developed to measure the percentage of successful detections (availability) obtained by each smartphone or tablet under these circumstances. Devices were placed on a tripod 1 m away from the beacon, making the microphones’ and transducer’s acoustic axis match at a 0.92 m height. Figure 6b shows the application interface during the test with one of the smartphones used.

**Figure 7:**
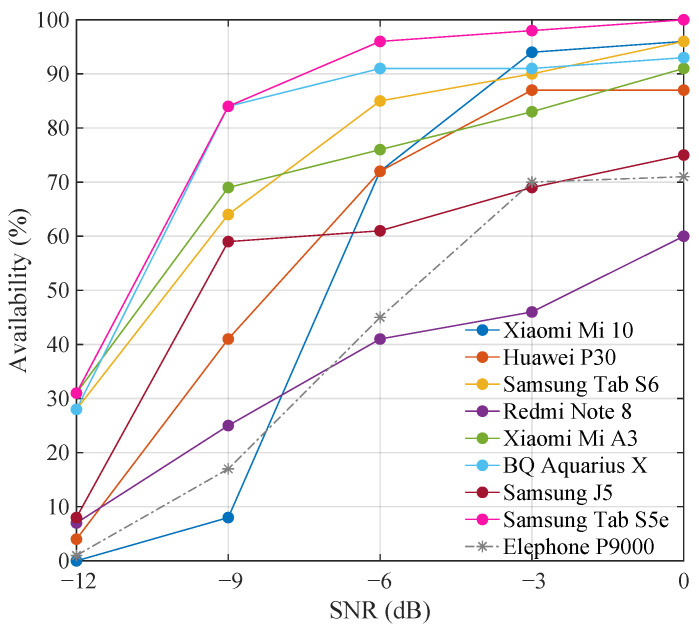
Devices availability against different signal to noise ratio.

All devices were tested, and their availability was measured for SNR of 0 dB, −3 dB, −6 dB, −9 dB, and −12 dB. The results obtained for each of these devices are detailed in Figure 7. The figure shows the performance differences offered by each of the smartphones and tablets that were used. On the right of the figure, it can be seen how the vast majority of these devices’ microphones offered sensitivities that provided a system availability above 70% for noise intensities equivalent to the emitted signal power, i.e., for an SNR of 0 dB. Only one device, the Xiaomi Redmi Note 8 Pro, offered slightly lower performance, making the availability decrease up to 60% under these conditions. However, it is worth mentioning that due to the wide variety of Android devices available in the market, it is possible to find devices that offer lower performance than the ones detailed here. By way of example, the results of the Elephone P9000 have been included. This phone’s microphone performance was considerably more flawed than the rest of the devices used, and although it managed to detect beacon emissions, it only did so for short ranges, as shown in Section 4.1.2. Therefore, although its results have been included to demonstrate its operation, performance comparison with the remaining devices could not be established. Regarding its use in the proposed PIPS, its only drawback was that users had to bring this device much closer to the emitting beacons.

To the left of the figure, it can be seen that as the power of AWGN increased, that is, the SNR decreased, all devices’ availability decreased to a greater or lesser extent. It can also be noted that for the lowest SNR value (−12 dB), there was a majority of devices whose availability fell below 10%. However, some still had an availability of around 30%. These results reveal that even in such challenging conditions of in-band noise addition as those carried out in this experiment, it was possible to identify the received signal even on devices with low sensitivity microphones thanks to the high coding process gain these signals have.

#### 4.1.2. Sensing Coverage

**Figure 8:**
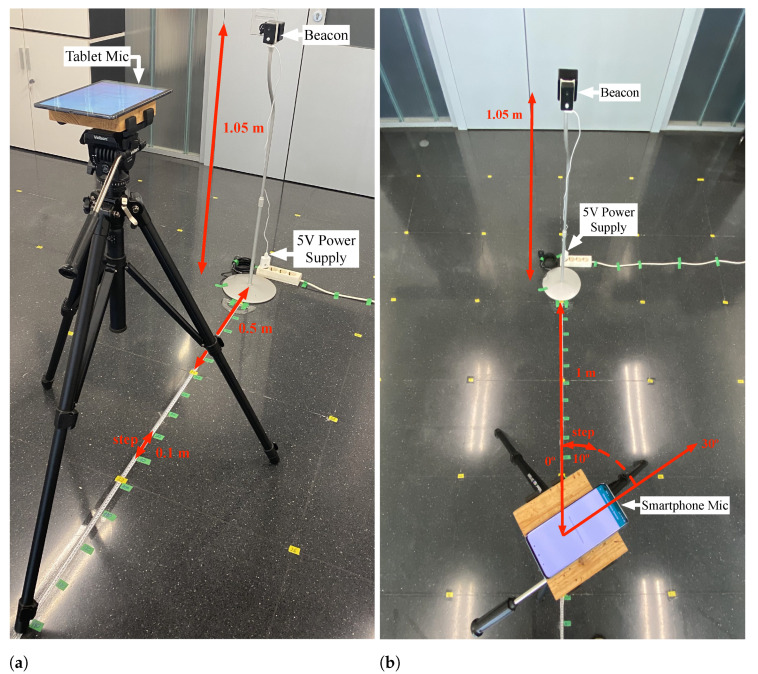
Sensing coverage experimental setups. (**a**) Device sensing with distance. (**b**) Device sensing with angle.

Each device’s detection capability was evaluated in the surroundings of the emitting beacon through two experimental tests. The assemblies of these experiments are detailed in Figure 8. This detection capability depended on the beacon’s emission power, on the directivity pattern, and the frequency response of its ultrasonic transducer. However, it also depended on the sensitivity and directivity pattern of each receiving device’s microphone. Therefore, to make a fair comparison between devices, the same emitting beacon was always used in all tests.

Figure 8a shows the experimental setup made to evaluate each device’s performance as the emitting beacon was moved away. For this study, the beacon’s transducer and the smartphone microphone acoustic axes were aligned, both located at the height of 1.05 m. Using the Android application, system availability measurements were taken for transmitter–receiver separations ranging from 0.1 m to 2.5 m in 0.1 m increments.

**Figure 9:**
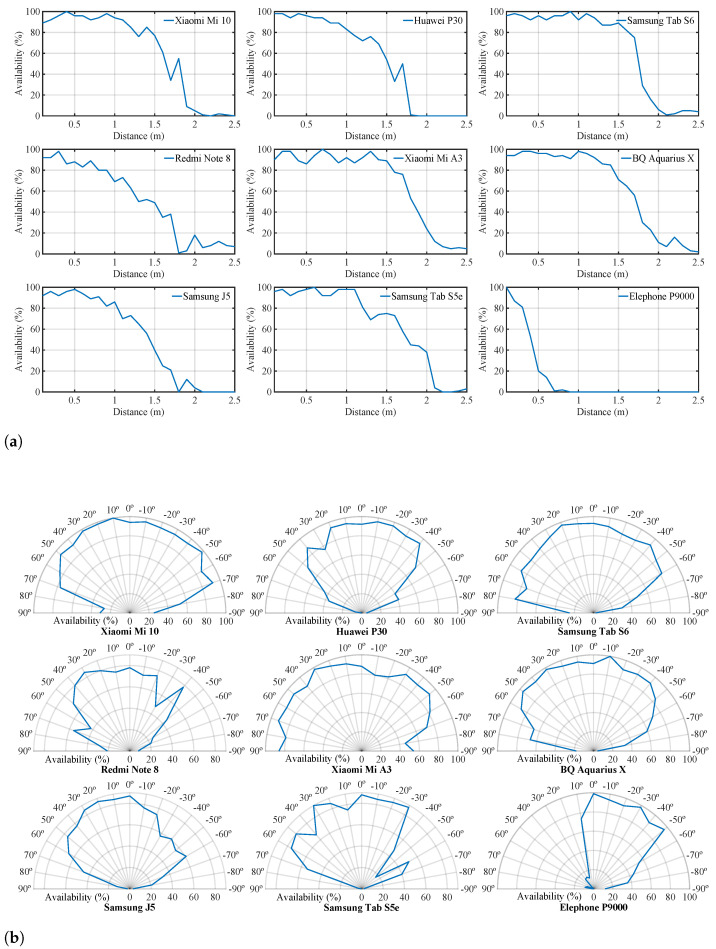
Sensing coverage experimental results. (**a**) Availability versus distance. (**b**) Availability versus angle at 1 m.

The results obtained in this experience are represented in Figure 9a. This figure shows how the system’s availability for practically all the devices was above 90% for beacon separation distances of less than 0.5 m. Between 0.5 m and 1 m, the vast majority of devices maintained an availability above 80%, except the Redmi Note 8 Pro, which fell to 70%. For distances between 1 m and 1.5 m, the drop in availability was more accentuated for some devices than for others. At 1.5 m, there were devices such as the Samsung Galaxy Tab S6, which maintained an availability close to 90%. However, other lower range devices such as the Samsung Galaxy J5 fell to 40%. Between 1.5 m and 2 m, all devices dropped below 40% availability, being the Samsung Galaxy Tab S5e, which maintained a higher availability with 38% at 2 m distance. Finally, between 2 m and 2.5 m, the availability of all devices decreased below 10%. The figure also includes the results obtained with the Elephone P9000. It can be seen that the low sensitivity of its microphone caused the system availability to drop quickly to 20% at a 0.5 m distance and reached 0% before 1 m separation with the beacon.

Likewise, Figure 8b shows the deployment made to determine the system availability depending on the emitter and receiver’s relative angle. The system availability was evaluated using the Android app every 10 degrees in a 1 m radius semicircle around the beacon. Specifically, from −90° to 90° at a 1-m distance from the beacon, where 0° matches the transducer acoustic axis. Figure 9b shows the results obtained in this experiment for each of the devices used. In this Figure, it can be seen that the results obtained on the devices’ microphone acoustic axis were above 80% availability, except for the Redmi Note 8 Pro, which was slightly below with an availability of 78%. These results were similar to the results obtained in the Figure 9a at the distance of 1 m. It can also be seen that there were three devices, Xiaomi Mi 10 Pro, Xiaomi Mi A3, and Samsung Galaxy Tab S6, that maintained this availability above 80% for signal reception angles between 60° and −60°. Other devices, such as the BQ Aquarius X and the Huawei P30, showed slightly lower performance, maintaining their availability at around 80% between angles of 50° and −50°. However, for this same angular range, devices such as the Samsung Galaxy Tab S5e, the Samsung Galaxy J5, and the Xiaomi Redmi Note 8 Pro offered lower performance with availabilities between 40–80%, generally performing better for positive angles. It can also be seen how for angles greater than ±60°, availabilities dropped sharply, falling below 20% at ±90° for all phones except the Xiaomi Mi A3. Finally, it can also be seen the Elephone P9000 had a minimal performance. Availability remained above 80% at close range for angles between 0° and −50°. However, it dropped sharply outside these limits.

### 4.2. Field Evaluation

Finally, a test of the system’s performance in a real scenario was conducted. For this purpose, a set of 18 beacons was deployed in an office building, and a route analyzing the signal detection performance of the different devices was made. Besides, a new Android application was designed to allow the user to know which beacon was detected associated with their position and that measured the decoding and detection times used to evaluate each mobile device’s performance. The decoding time $t_{d}$ was the mobile phone or tablet’s time to process the received signal and obtain the associated code. This time was a roughly constant parameter for each device in the absence of other processes and was related to its computational capacity. Moreover, the beacon detection time $t_{b}$ was when the device detected the same code twice in a row. This redundancy measure was taken as a precaution to avoid false positive detection when many beacons were deployed. Consequently, this time was related to the quality of the emitted signal and the sensitivity of each device’s microphone, increasing the time in those cases where the conditions for detection were not optimal. Additionally, the distance $r_{b}$ at which each detection occurred was measured with a laser rangefinder [58] and recorded to evaluate the different locations’ coverage along the path.

**Figure 10:**
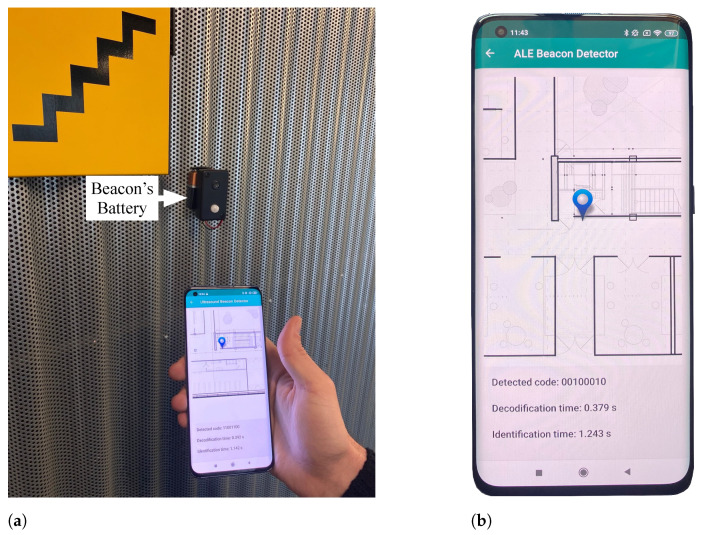
Field evaluation experiment. (**a**) Field evaluation experimental setup. (**b**) Android application interface.

In Figure 10, on the left Figure 10a shows a mobile phone with the application detecting one of the beacons located next to a staircase. In this figure, it can also be appreciated that a 9 V battery powered the beacon. On the right, Figure 10b shows the application screenshot displaying the building’s plan, and highlighting the beacon that was just detected. Besides, the detected code together with $t_{d}$ and $t_{b}$ time values are provided. A demonstrative video of the system operation can be found in the Supplementary Materials at the end of the paper.

Table 1 presents the results obtained for $t_{b}$ and $r_{b}$ with the nine devices at each of the 18 beacons deployed along the trajectory. Firstly, this table indicates the best mean values of these parameters in green, with the absolute best value of each highlighted in bold. The worst mean values of these parameters are indicated in red, with their absolute worst values also marked in bold. This table shows that all the devices had beacon detection times $t_{b}$ of around 1 s. The $t_{b}$ absolute best value was 1.036 s, achieved by the Xiaomi Mi 10 Pro, thanks to its faster processing time. Concerning the mean $t_{b}$ values, the Xiaomi Mi 10 again showed the best performance. For the rest of the devices, we can see that most of them did not exceed a $t_{b}$ value above 2 s. However, four devices exceeded this threshold: the Huawei P30 and the Samsung Galaxy Tab S5e both exceeded it by a small margin, while the Samsung Galaxy J5 exceeded it by a broader range with 3.420 s, and the Elephone P9000 reached 3.660 s. The latter device also marks a $t_{b}$ absolute worst value with 8.754 s.

Furthermore, it can be seen how these last two devices had high Standard Deviations (SD) values that were motivated by the low performance of these mid-range devices that were also more than 4 years old. In summary, it can be seen how the proposed system generally offered detection times close to one second, with delays of up to 2 s in some models of phones with lower performance. Exceptionally, delays between 3 and 9 s could be obtained in extreme cases with older, low-performance devices.

Referring to the $r_{b}$ values, they ranged from a worst value of 0.210 m for the Elephone P9000 due to its lowest microphone sensitivity to a best value of 3.080 m obtained by the Xiaomi Mi A3, which was confirmed to be the device with the best microphone performance. This phone also marked the best average $r_{b}$ value with 1.762 m. Regarding the other devices’ mean values, five of them, the Xiaomi Mi 10 Pro, the Huawei P30, the Samsung Galaxy Tab S6, the Redmi Note 8 Pro, and the Xiaomi Mi A3, offered $r_{b}$ values above one meter. However, there were four other devices, the BQ Aquarius X, the Galaxy Tab S5e, the Galaxy J5, and the Elephone P9000, that provide $r_{b}$ distances below one meter. The latter device, the Elephone P9000, obtained the $r_{b}$ worst mean and also the absolute worst value with 0.335 m and 0.210 m, respectively. About this parameter’s SD values, it is worth noting that all devices offered non-negligible results. These mean’s dispersions were related not only to the device performance but also to the acoustic environment where each of the beacons was located. For instance, *Beacon 18* had maximum $r_{b}$ values for three devices and a minimum $t_{b}$ value for another one. These results could be a sign that this beacon was in a more favorable acoustic environment than others.

Table 2 shows the measured results for the mean decoding time ${\bar{t}}_{d}$ and the standard deviation σ for each device. The table also shows the worst values of each parameter in red and the best values in green. As can be seen, ${\bar{t}}_{d}$ was related to the computational speed of each device. As known, the device with the highest processing power was the Xiaomi Mi 10 Pro, a new high-end device. This smartphone only required an average time of 0.387 s to decode the signals. Xiaomi Mi 10 was also the most stable device when running the decoding algorithm as it also had the lowest SD with only 0.018 s.

Regarding the rest of devices, three of them belonging to the upper-middle-range, the Huawei P30, the Samsung Galaxy Tab S6, and the Redmi Note 8 Pro, showed decoding times of around 0.5 s, with standard deviations below 0.05 s. Other devices such as the BQ Aquarius X and the Samsung Galaxy Tab S5e were older mid-range devices with slightly lower performance providing decoding times around 0.65 s and SD of 0.048 s and 0.079 s, respectively. The next tier was the Xiaomi Mi A3 and the Samsung Galaxy J5 with ${\bar{t}}_{d}$ values of 0.814 s and 0.951 s. Both devices featured a mid-range hardware. However, the Xiaomi Mi A3 had a more up-to-date processor, offering subtly better performance. These devices’ SDs were also low, with values of 0.045 s and 0.071 s, respectively. Finally, the Elephone P9000 was the device that offered the highest processing time with 1.105 s. It also had the highest variability, offering a substantial SD value of 0.214 s.

### 4.3. Energy Efficiency

In this subsection, a study of the proposed system’s energy efficiency was carried out. With this purpose, we evaluated the energy consumption of both the beacons and each receiver. Since these beacons were designed to have a low energy consumption, the experimental evaluation of their autonomy is a long-term process that is still ongoing. However, it was possible to provide a theoretical estimate of the beacon’s power consumption and consequent autonomy.

A very low power consumption microcontroller was used for these beacons’ implementation. According to the manufacturer’s specifications [59], this device required a power consumption of 84 µA/MHz in run mode. As the clock was set to 48 MHz, this implied a consumption of 4 mA. By contrast, the device only consumed 28 nA in standby mode. Besides, during beacon operation, it was necessary to power the audio amplifier, which according to its manufacturer [47], had a consumption of 4 mA. Finally, the PIR sensor [48] also required a 0.02 mA current. In theory, this meant that when the beacon was in active mode, i.e., emitting because it had detected the user’s presence, it required 4 mA + 4 mA + 0.02 mA = 8.02 mA. However, when the beacon was in standby mode, the consumption was reduced to 0.02 mA + 0.000028 mA ≈ 0.02 mA.

To enable the microcontroller’s low power mode, it was necessary to supply the NUCLEO-L432KC board through its 3V3 pin, and remove two tiny Soldering Bridges (SB) labelled by the manufacturer as SB9 and SB14. Once this low power setting was established, the board could not be programmed any more. The NUCLEO-L432KC board and PIR sensor were powered directly from two AA batteries. The batteries also had to power the audio amplifier which had too high consumption to remain always on. This problem could be solved using the PIR sensor signal to simultaneously wake up the microcontroller and drive the base of a C547B bipolar transistor [60] acting as a switch for the amplifier power supply. This latter setup had an experimentally measured power consumption of 11.8 mA in operation mode and 0.02 mA in standby mode. Real beacons consumption was been evaluated for 2 months by placing them in different areas of the laboratory building. During this period, the total number of emissions per beacon was collected. This number depends on where the beacon was located, obtaining an average value of 140 emissions per day in the busiest locations. Considering that each emission took 2.5 s, the beacons consumed (2.5/3600)×140×11.8=1.15 mAh in daily emissions. If the standby consumption for the rest of the day was also added (24−(2.5×140)/3600)×0.02 = 0.48 mAh, a total daily consumption of 1.15 + 0.48 = 1.63 mAh was obtained. With this estimated consumption, the ALE beacon could operate for 4.2 years using two AA alkaline batteries with 2500 mAh capacity.

If the ALE beacon consumption was compared with that of a commercial BLE beacon such as the Accent Systems iBKS [61], it could be concluded that both beacons had a similar performance. These BLE beacons were based on the nRF51822 chipset [62], which had an estimated power consumption of 10.5 mA transmitting at a power of 4 dB. This consumption was only 11% lower than that required by the ALE beacon.

Regarding each Android device’s consumption during beacon detection, a comparison with BLE beacon scan was conducted. This comparison is based on the fact that BLE couldcan be considered the reference technology in PIPS and LBS development nowadays. BLE beacons’ consumption was low and they could operate for years with the same batteries, but on the other hand their scanning required much energy from the receivers.

**Figure 11:**
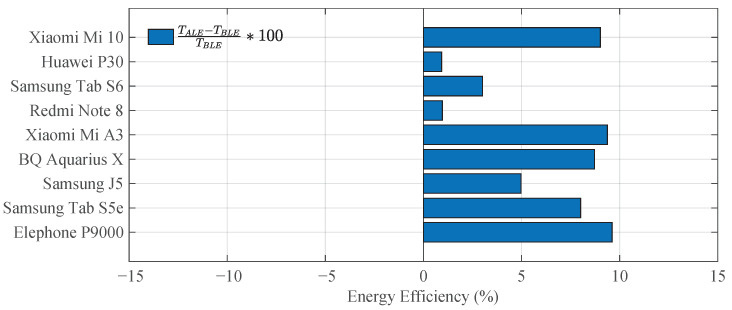
Energy balance of Bluetooth Low Energy (BLE) and Acoustic Low Energy (ALE) beacon scanning with each device.

To evaluate both consumptions, an Android application was designed to scan for BLE or ALE beacons continuously. The application also recorded the remaining battery percentage every 5 minutes until the device’s battery was completely depleted. First, all devices were fully charged and then, the Android app was configured to scan for BLE beacons, letting it run until the devices drained their batteries. The same procedure was repeated using the ALE beacon detection algorithm. In both experiments, the displays remained dimly lit to simulate a regular operation. The results obtained in this experiment can be seen in Figure 11 where $T_{ALE}$ and $T_{BLE}$ represent the battery life when exploring ALE and BLE beacons respectively.

These results showed the relative increase in battery life when exploring ALE instead of BLE beacons. As can be noticed, in all cases this increment was positive, ranging from a minimum of 2% for the Huawei P30 and the Redmi Note 8 to a maximum of 9% for other devices, such as the Xiaomi Mi 10, the Xiaomi Mi A3, or the Elephone P9000. These results demonstrated that exploring ALE beacons had a lower energy cost for the users’ devices than scanning BLE beacons.

## 5. Conclusions

In this work, the design of an Acoustic Low Energy (ALE) beacon has been proposed to develop a Proximity-Based Indoor Positioning System (PIPSs). This beacon is constituted by low power consumption components and it is also equipped with a PIR sensor that optimizes energy saving by emitting only in the presence of a user. The emission scheme has been specially developed to use very low amplitude inaudible signals, which can still be detected in hostile acoustic conditions with intense in-band noise. These emissions use Linearly Frequency Modulated (LFM) signals to encode 8-bit messages that are detected by pulse compression. The number of bits has been set to *n* = 8, although it can be easily scaled up to tag $2^{n}$ different beacons.

A set of experimental tests have been carried out with nine Android devices to evaluate the system performance. First, the system’s noise tolerance has been evaluated by generating additive white Gaussian noise (AGWN) next to the emitting beacon, and measuring the percentage of successful detections (Availability) in each device for different levels of this noise. The results have revealed that most devices continue to detect with Signal to Noise Ratios (SNRs) as low as −12 dB.

Next, the microphone sensitivity of each device has been evaluated. For this purpose, its availability has been measured depending on the distance and the bearing to the transmitting beacon. These results have demonstrated that, except for one device with low performance, the remaining ones have an availability above 80% at a 1-m distance from the transmitting beacon. With respect to bearing, a majority of devices have an availability above 80% at 1 meter from the beacon for angles up to 50° with respect to the beacon’s acoustic axis.

Additionally, the system behavior has been evaluated in a real scenario. For this purpose, a Proximity-Based Indoor Positioning System (PIPS) has been deployed with a total of 18 beacons inside an office building. In this study, it has been demonstrated that all devices feature an average code identification time of around one second. Besides, it has been found that those devices with better microphone sensitivities have been able to detect beacons up to distances of 3 m in the best case.

Finally, the ALE beacon’s energy consumption during a typical operation has been estimated, turning out to be comparable to that of some Bluetooth Low Energy (BLE) beacons available in the market. Additionally, a power consumption comparison between the ALE beacon detection algorithm and the BLE beacon scan operation has been carried out with each receiving device. In this comparison, it has been demonstrated that the ALE beacon detection algorithm is more energy-efficient than the BLE beacon scan operation in all cases, reaching a relative increment of battery life that ranges from 2% to a maximum of 9%.

## Figures and Tables

**Table 1:** Beacon detection time $t_{b}$ (s) and detection range $r_{b}$ (m) for each device and code. Each parameter’s best values are in bold green, and the worst values are in bold red.

	Mi 10		P30		Tab S6		Note 8		Mi A3		BQ		J5		Tab S5e		P9000	
Beacon	*t_b_* (s)	*r_b_* (m)	*t_b_* (s)	*r_b_* (m)	*t_b_* (s)	*r_b_* (m)	*t_b_* (s)	*r_b_* (m)	*t_b_* (s)	*r_b_* (m)	*t_b_* (s)	*r_b_* (m)	*t_b_* (s)	*r_b_* (m)	*t_b_* (s)	*r_b_* (m)	*t_b_* (s)	*r_b_* (m)
1	1.072	0.682	1.648	0.761	1.251	0.423	1.225	0.951	1.436	1.218	1.153	0.462	3.519	0.425	1.716	0.460	2.901	0.383
2	1.065	1.471	2.079	0.625	1.276	1.393	1.573	0.633	1.822	1.955	1.233	1.181	1.996	0.713	2.215	1.625	1.936	0.362
3	1.059	0.773	4.57	1.225	1.471	1.411	3.121	1.152	1.089	0.720	1.377	0.925	4.109	0.546	3.124	0.860	1.909	0.457
4	1.039	1.290	1.586	1.372	1.694	1.124	1.039	0.706	3.594	**3.080**	1.183	1.04	5.980	1.080	3.168	1.120	1.276	0.410
5	1.106	1.387	3.605	0.847	1.848	1.292	1.039	1.135	1.117	0.973	1.240	0.954	1.987	0.937	2.745	0.780	4.017	0.484
6	1.061	1.035	1.274	1.268	1.273	1.520	1.580	1.385	3.141	1.900	1.173	1.103	4.804	0.711	6.512	1.184	**8.754**	0.356
7	1.145	0.970	1.896	1.281	1.201	1.680	1.262	0.650	1.132	2.010	1.697	1.03	1.805	0.626	1.251	1.340	6.268	0.314
8	1.067	1.547	1.451	1.004	1.239	0.715	1.085	0.594	2.381	1.580	2.554	0.841	2.054	0.598	6.735	0.889	5.900	0.257
9	1.208	1.239	1.481	1.190	1.049	1.134	1.820	0.670	1.144	2.678	1.230	1.129	1.803	1.559	1.533	0.682	8.571	0.332
10	1.070	0.648	2.077	0.948	1.158	0.857	1.104	0.948	4.179	1.216	1.218	0.455	5.126	0.706	1.144	0.675	3.725	**0.210**
11	1.832	0.922	1.250	1.503	1.086	1.524	1.327	1.789	1.191	1.864	1.787	0.787	5.022	0.705	1.137	1.075	6.490	0.301
12	1.701	1.023	2.227	0.863	1.346	0.991	1.085	1.473	1.132	2.820	1.214	0.972	1.522	2.131	2.162	1.21	1.933	0.330
13	**1.036**	0.644	1.091	1.402	1.223	0.803	1.133	1.362	1.122	1.616	1.238	1.059	1.411	0.624	1.111	0.923	1.667	0.253
14	1.083	1.419	1.084	0.977	1.196	0.690	1.119	0.567	1.061	1.082	2.192	1.017	6.109	0.559	1.137	0.270	1.629	0.334
15	1.079	0.553	2.586	1.351	1.078	0.705	1.075	1.229	1.185	1.627	1.238	0.589	6.059	0.421	1.248	0.592	1.872	0.292
16	1.071	1.220	2.762	1.636	1.139	0.403	3.124	2.819	1.168	1.905	1.163	1.22	4.003	1.047	1.132	0.463	2.061	0.287
17	1.048	0.524	1.580	1.413	1.226	0.966	1.105	1.742	1.164	1.484	1.188	0.478	5.113	0.453	1.070	0.546	3.005	0.261
18	1.071	1.656	2.071	1.647	1.063	1.074	1.048	1.546	2.311	2.072	1.326	1.148	2.032	0.274	1.227	0.954	1.701	0.466
Mean	**1.156**	1.053	2.018	1.180	1.231	1.038	1.437	1.177	1.742	**1.762**	1.411	0.910	3.420	0.678	2.242	0.869	**3.660**	**0.335**
σ	**0.226**	0.363	0.908	0.301	0.283	0.378	0.651	0.566	0.976	**0.635**	0.395	0.127	1.743	0.317	1.742	0.349	**2.451**	**0.077**

**Table 2:** Beacon decoding time $t_{d}$ (s) and its standard deviation σ (s) for each device.Each parameter’s best values are in bold green, and the worst values are in bold red.

	Mi 10	P30	Tab S6	Note 8	Mi A3	BQ	J5	Tab S5e	P9000
${\bar{t}}_{d}$ (s)	**0.387**	0.508	0.499	0.541	0.814	0.658	0.951	0.659	**1.105**
σ (s)	**0.018**	0.022	0.047	0.050	0.045	0.048	0.071	0.079	**0.214**

## Supplementary Materials

The following is available online at https://www.mdpi.com/1424-8220/21/5/1703/s1, Video S1: Demonstration of system operation.

## References

[1] Mautz R. (2012). Indoor Positioning Technologies. Ph.D. Thesis.

[2] Brena R.F., García-Vázquez J.P., Galván-Tejada C.E., Muñoz-Rodriguez D., Vargas-Rosales C., Fangmeyer J. (2017). Evolution of Indoor Positioning Technologies: A Survey. J. Sens..

[3] Mendoza-Silva G.M., Torres-Sospedra J., Huerta J. (2019). A Meta-Review of Indoor Positioning Systems. Sensors.

[4] He S., Chan S.G. (2016). Wi-Fi Fingerprint-Based Indoor Positioning: Recent Advances and Comparisons. IEEE Commun. Surv. Tutorials.

[5] Torres-Sospedra J., Richter P., Moreira A., Mendoza-Silva G., Lohan E.S., Trilles S., Matey-Sanz M., Huerta J. (2020). A comprehensive and reproducible comparison of clustering and optimization rules in wi-fi fingerprinting. IEEE Trans. Mob. Comput..

[6] Faragher R., Harle R. (2015). Location Fingerprinting With Bluetooth Low Energy Beacons. IEEE J. Sel. Areas Commun..

[7] Aranda F.J., Parralejo F., Álvarez F.J., Torres-Sospedra J. (2020). Multi-Slot BLE Raw Database for Accurate Positioning in Mixed Indoor/Outdoor Environments. DATA.

[8] Lee J., Kim S., Han S. (2019). 3D Visible Light Indoor Positioning by Bokeh Based Optical Intensity Measurement in Smartphone Camera. IEEE Access.

[9] Amsters R., Holm D., Joly J., Demeester E., Stevens N., Slaets P. (2020). Visible Light Positioning Using Bayesian Filters. J. Light. Technol..

[10] Murano S., Pérez-Rubio C., Gualda D., Álvarez F.J., Aguilera T., Marziani C.D. (2020). Evaluation of Zadoff–Chu, Kasami, and Chirp-Based Encoding Schemes for Acoustic Local Positioning Systems. IEEE Trans. Instrum. Meas..

[11] Aparicio J., Aguilera T., Álvarez F.J. (2020). Robust Airborne Ultrasonic Positioning of Moving Targets in Weak Signal Coverage Areas. IEEE Sens. J..

[12] Basiri A., Lohan E.S., Moore T., Winstanley A., Peltola P., Hill C., Amirian P., Figueiredo e Silva P. (2017). Indoor location based services challenges, requirements and usability of current solutions. Comput. Sci. Rev..

[13] Ni L.M., Liu Y., Lau Y.C., Patil A.P. LANDMARC: Indoor location sensing using active RFID. Proceedings of the First IEEE International Conference on Pervasive Computing and Communications (PerCom 2003).

[14] Montaser A., Moselhi O. (2014). RFID indoor location identification for construction projects. Autom. Constr..

[15] Ozdenizci B., Ok K., Coskun V., Aydin M.N. Development of an Indoor Navigation System Using NFC Technology. Proceedings of the 2011 Fourth International Conference on Information and Computing.

[16] Goronzy G., Pelka M., Hellbrück H. QRPos: Indoor positioning system for self-balancing robots based on QR codes. Proceedings of the 2016 International Conference on Indoor Positioning and Indoor Navigation (IPIN).

[17] Chawathe S.S. (2009). Low-latency indoor localization using bluetooth beacons. Proceedings of the 2009 12th International IEEE Conference on Intelligent Transportation Systems.

[18] Fazio M., Buzachis A., Galletta A., Celesti A., Villari M. A proximity-based indoor navigation system tackling the COVID-19 social distancing measures. Proceedings of the 2020 IEEE Symposium on Computers and Communications (ISCC).

[19] Kouyoumdjieva S.T., Karlsson G. Experimental Evaluation of Precision of a Proximity-based Indoor Positioning System. Proceedings of the 2019 15th Annual Conference on Wireless On-demand Network Systems and Services (WONS).

[20] Radhakrishnan M., Misra A., Balan R.K., Lee Y. Smartphones and BLE Services: Empirical Insights. Proceedings of the 2015 IEEE 12th International Conference on Mobile Ad Hoc and Sensor Systems.

[21] Cho K., Park W., Hong M., Park G., Cho W., Seo J., Han K. (2015). Analysis of Latency Performance of Bluetooth Low Energy (BLE) Networks. Sensors.

[22] Álvarez F.J. (2019). Fundamentals of Airborne Acoustic Positioning Systems. Geographical and Fingerprinting Data to Create Systems for Indoor Positioning and Indoor/Outdoor Navigation.

[23] Boucheron R. Over-sampling improvement for acoustic triangulation using Barker code audio signals. Proceedings of the 173rd Meeting of Acoustical Society of America and 8th Forum Acusticum.

[24] Ureña J., Hernández Á., García J.J., Villadangos J.M., Carmen Pérez M., Gualda D., Álvarez F.J., Aguilera T. (2018). Acoustic Local Positioning With Encoded Emission Beacons. Proc. IEEE.

[25] Paredes J.A., Aguilera T., Álvarez F.J., Lozano J., Morera J. (2011). Analysis of Doppler Effect on the Pulse Compression of Different Codes Emitted by an Ultrasonic LPS. Sensors.

[26] Aguilera T., Álvarez F.J., Paredes J.A., Moreno J.A. (2020). Doppler compensation algorithm for chirp-based acoustic local positioning systems. Digit. Signal Process..

[27] Lin H., Liu G., Li F., Zuo Y. (2021). Where to go? Predicting next location in IoT environment. Front. Comput. Sci..

[28] Edwan E., Bourimi M., Joram N., Al-Qudsi B., Ellinger F. NFC/INS integrated navigation system: The promising combination for pedestrians’ indoor navigation. Proceedings of the 2014 International Symposium on Fundamentals of Electrical Engineering (ISFEE).

[29] Pecchioli L., Pucci M., Mohamed F., Mazzei B. Browsing in the virtual museum of the sarcophagi in the Basilica of St.Silvestro at the Catacombs of Priscilla in Rome. Proceedings of the 2012 18th International Conference on Virtual Systems and Multimedia.

[30] Al-Saedi S.B., Azim M.M.A. Radio Frequency Near Communication (RFNC) Technology: An Integrated RFID-NFC System for Objects’ Localization. Proceedings of the 2017 9th IEEE-GCC Conference and Exhibition (GCCCE).

[31] Tesoriero R., Tebar R., Gallud J., Lozano M., Penichet V. (2010). Improving location awareness in indoor spaces using RFID technology. Expert Syst. Appl..

[32] Rahim M., Rahman M., Rahman M., Asyhari A.T. (2021). andBhuiyan, M.; Ramasamy, D. Evolution of IoT-enabled connectivity and applications in automotive industry: A review. Veh. Commun..

[33] Amutha B., Nanmaran K. Development of a ZigBee based virtual eye for visually impaired persons. Proceedings of the 2014 International Conference on Indoor Positioning and Indoor Navigation (IPIN).

[34] Xiao L., Yan Q., Lou W., Chen G., Hou Y.T. (2013). Proximity-Based Security Techniques for Mobile Users in Wireless Networks. IEEE Trans. Inf. Forensics Secur..

[35] Mackey A., Spachos P., Plataniotis K.N. (2020). Smart Parking System Based on Bluetooth Low Energy Beacons With Particle Filtering. IEEE Syst. J..

[36] Spachos P., Plataniotis K.N. (2020). BLE Beacons for Indoor Positioning at an Interactive IoT-Based Smart Museum. IEEE Syst. J..

[37] Ceron J.D., Lopez D.M., Ramirez G.A. (2017). A mobile system for sedentary behaviors classification based on accelerometer and location data. Comput. Ind..

[38] Xie C., Guan W., Wu Y., Fang L., Cai Y. (2018). The LED-ID Detection and Recognition Method Based on Visible Light Positioning Using Proximity Method. IEEE Photonics J..

[39] Kim M., Suh T. (2019). A Low-Cost Surveillance and Information System for Museum Using Visible Light Communication. IEEE Sens. J..

[40] Shahid B., Kannan A.A., Lovell N.H., Redmond S.J. Ultrasound user-identification for wireless sensor networks. Proceedings of the 2010 Annual International Conference of the IEEE Engineering in Medicine and Biology.

[41] Rossi M., Seiter J., Amft O., Buchmeier S., Tröster G. (2013). RoomSense: An Indoor Positioning System for Smartphones Using Active Sound Probing. Proceedings of the 4th Augmented Human International Conference.

[42] Jia R., Jin M., Chen Z., Spanos C.J. (2015). SoundLoc: Accurate room-level indoor localization using acoustic signatures. Proceedings of the 2015 IEEE International Conference on Automation Science and Engineering (CASE).

[43] Hammoud A., Deriaz M., Konstantas D. Robust ultrasound-based room-level localization system using COTS components. Proceedings of the 2016 Fourth International Conference on Ubiquitous Positioning, Indoor Navigation and Location Based Services (UPINLBS).

[44] Minkoff J. (1992). Signals, Noise and Active Sensors: Radar, Sonar, Laser Radar.

[45] Álvarez F.J., Aguilera T., López-Valcarce R. (2017). CDMA-based acoustic local positioning system for portable devices with multipath cancellation. Digit. Signal Process..

[46] STM32 Nucleo-32 Development Board with STM32L432KC MCU. https://www.st.com/en/evaluation-tools/nucleo-l432kc.html.

[47] Adafruit Class D Audio Amplifier 2.5 W. https://cdn-shop.adafruit.com/datasheets/PAM8302A.pdf.

[48] MH-ET LIVE Pasive Infrared Sensor. https://forum.mhetlive.com/topic/46/mh-et-live-sr-602-pyroelectric-human-infrared-sensor-module.

[49] RS Amidata Ultrasonic Transducer. https://docs.rs-online.com/c0bf/0900766b816c0809.pdf.

[50] GRAS 40BE 1/4” Ultrasonic Microphone. https://www.gras.dk/products/measurement-microphone-cartridge/prepolarized-cartridges-0-volt/product/158-40be.

[51] GRAS 12AK 1-Channel Power Module. https://www.gras.dk/products/power-module/product/225-12ak.

[52] Agilent 33522A Arbitrary Waveform Generator. https://www.keysight.com/en/pd-1871286-pn-33522A/function-arbitrary-waveform-generator-30-mhz?cc=US&lc=eng.

[53] Klauder J.R., Price A.C., Darlington S., Albersheim W.J. (1960). The theory and design of chirp radars. Bell Syst. Tech. J..

[54] AudioRecord Public Class Android. https://developer.android.com/reference/android/media/AudioRecord.

[55] Piotr Wendykier jTransforms Open Source Library. https://sites.google.com/site/piotrwendykier/software/jtransforms.

[56] Allen J.B., Berkley D.A. (1979). Image method for efficiently simulating small-room acoustics. J. Acoust. Soc. Am..

[57] Tektronix TDS 2004C Oscilloscope. https://uk.tek.com/oscilloscope/tds2000-digital-storage-oscilloscope.

[58] Rangefinder GLM 80 Bosch. https://www.bosch-professional.com/gb/en/products/glm-80-0601072370.

[59] Microcontrollers Energy Consumption STM32L4. https://www.st.com/resource/en/application_note/dm00216518-optimizing-power-and-performance-with-stm32l4-series-microcontrollers-stmicroelectronics.pdf.

[60] C547B NPN Transistor Fairchild Semiconductor. https://docs.rs-online.com/4ff8/0900766b812cf5d7.pdf.

[61] iBKS Bluetooth Low Energy Beacon Accent Systems. https://accent-systems.com/product/ibks-plus/?v=04c19fa1e772.

[62] nRF51822 Nordic Semiconductor. https://www.nordicsemi.com/-/media/Software-and-other-downloads/Product-Briefs/nRF51822-product-brief.pdf?la=en&hash=A4B5A9AA6675A58F7B779AF81C860CD69EB867FD.

